# Epigenetic Regulation of B Cell Memory Formation: A Poised Model for B Cell Epigenetic Reprograming

**DOI:** 10.1155/jimr/9328523

**Published:** 2025-07-17

**Authors:** Carlos Valverde-Hernandez

**Affiliations:** Nuffield Department of Surgical Sciences, John Radcliffe Hospital, University of Oxford, Oxford OX3 9DU, UK

**Keywords:** B cell fate determination, B cell memory, epigenetics, germinal center, memory B cells, poised genes

## Abstract

The formation of B cell immunological memory happens after the first encounter with a pathogen. At the germinal center (GC), B cells experience complex transcriptional and epigenetic transitions to differentiate into memory B cells (MBCs) and plasma cells (PCs). In particular, the differentiation of GC B cells into MBCs has been poorly understood, and no clear conclusions on the signals and transcription factors leading to this cell fate have been identified. Recent discoveries in epigenetics and immune memory have elucidated the essential role of epigenetic regulators in establishing the memory B cell (MBC) fate. DNA methylation regulators, histone modifiers, noncoding RNAs (ncRNAs), and chromatin remodelers orchestrate a dynamic reprograming of the MBC phenotype. Positive and negative epigenetic regulators of the B cell program collaborate at each differentiation stage and allow for complex chromatin topology rearrangements and dynamic exposure to transcription and translation. Following MBC fate determination at the GC, the acquired epigenetic modifications induce a poised regulatory state where genes are epigenetically marked to remain transcriptionally inactive, but primed for rapid activation upon stimuli. Thus, a poised epigenetic control over gene expression governs MBC formation and a novel model of epigenetic reprograming is proposed. This model provides a novel perspective on how the B cell fate is determined in the GC and memory is formed, offering insights for improved vaccination and therapeutical approaches.

## 1. Introduction

The adaptive immune response is mediated by a diverse repertoire of cellular and humoral components specific to discrete antigens. This level of specificity is only acquired following a series of highly regulated processes by which T and B cells develop, differentiate, and adapt their receptors and function. Pathogen recognition by antigen-specific lymphocytes generates immunological memory, providing a rapid and enhanced response upon pathogen reexposure [1]. To this end, T and B lymphocytes need to experience important genetic and epigenetic changes from precursor to effector lymphocyte states [2, 3]. Thus, an efficient coordination of transcriptional states and epigenetic reprograming is required to establish memory at the cellular level [4]. Given that epigenetics is the study of heritable changes in the DNA, it has the potential to unravel the mechanisms underlying the plasticity of lymphocyte memory regulators [4]. Hence, focusing on the epigenetic control and reprograming of lymphocyte gene expression is a matter of importance in the understanding of adaptive immunity and memory acquisition.

B cells or B lymphocytes initially develop in the bone marrow, and migrate to peripheral lymphoid organs where they mature and differentiate. It is then at germinal centers (GCs) where they undergo affinity-driven clonal expansion and selection into GC-dependent memory B cells (MBCs) and long-lived plasma cells (PCs) [5, 6]. Among multiple functions, B cells are responsible for generating high affinity, antigen-specific antibodies establishing the basis of the humoral immune response [5]. Analogously, B cell memory formation is key to pathogen recognition, response, and reinfection [7]. GC-dependent MBCs are described to be low affinity long-lived B cells, exported from the GC reaction and capable to reactivate from a quiescent state after antigen reexposure [6, 7]. Hence, MBCs require high plasticity to allow for complex gene expression alterations. Conversely, plasmablasts (PBs) and long-lived PCs are high affinity products of the GC with enhanced antibody production activity [5, 6]. Furthermore, GC-independent MBCs and short-lived PCs can be formed upon cognate antigen recognition [6, 8]. The primary focus of this review will be GC-dependent B cell memory formation, the most frequent and studied form of immunological B cell memory.

The selection mechanisms leading to the MBC fate as opposed to PB differentiation or GC recycle are only partially understood, and different models have been proposed [9]. In the last decades, the molecular understanding of MBC differentiation has increased, and potential transcription regulators have been described [9]. Nevertheless, it remains unclear whether there is a positive signal or a “master” transcription regulator of MBC fate determination [8, 9]. Factors such as the increasing affinity to antigen through B cell receptor (BCR) signaling, the time B cells spend at the GC, or the strength of T follicular helper (Tfh) cell support to circulating GC B cells are studied mechanisms contributing to B cell selection and division [5, 9]. Similarly, the epigenetic control of GC B cells is suggested to play an important role in MBC differentiation, potentially adding a new dimension to the current paradigm [9]. Here, it is proposed that a “poised epigenetic control” of the selection steps occurring at the GC, referred to as the dynamic chromatin state characterized by coexisting active and repressive epigenetic marks enabling genes to remain transcriptionally inactive yet primed for activation during differentiation, is critical for B cells to differentiate into MBCs as opposed to PCs, GC reentry or apoptosis. In this review, the epigenetic mechanisms controlling B cell memory formation are discussed, the major transcriptional and epigenetic regulators of MBC selection contributing to a poised epigenetic landscape are evaluated, and a novel governing model of epigenetic reprograming in B cell memory formation is proposed.

### 1.1. Mechanisms of Epigenetic Regulation of B Cells

Different mechanisms of epigenetic control orchestrate the GC reaction, and shape the MBC phenotype by influencing gene transcription and chromatin accessibility. Hitherto, epigenetic events have been able to explain processes, such as antibody V(D)J recombination (variable, diversity, and joining immunoglobulin genes), somatic hypermutation (SHM), and class switch recombination (CSR) [10, 11]. This knowledge raised from elucidating the strict requirement for chromatin accessibility needed for recombination-activating genes (RAG1 and RAG2) (in V(D)J recombination) or activation-induced cytidine deaminase (AID) (in SHM and CSR) interaction with specific DNA loci [10, 11]. However, these B cell mechanisms have been extensively reviewed by Li et al. [11] and later complemented by Moroney et al. [12] and will not be discussed in this review. Similarly, DNA methylation, histone modifications, noncoding RNA regulation, and other higher-tier chromatin structure rearrangements have been described to regulate B cell memory formation. Therefore, the epigenetic mechanisms controlling the establishment of the GC B cell phenotype and regulating its differentiation into MBCs and PCs are central to define the formation of immunological memory by B cells. These epigenetic mechanisms, summarized in Table 1, together establish a primed chromatin state by which poised genes can be rapidly switched from a transcriptionally repressed to an activated state. As a consequence, MBCs can promptly undergo a phenotypic switch towards increased metabolic demands, further differentiation into PCs, and clonal expansion upon antigen reencounter.

### 1.2. Epigenetic Control of B Cell Memory by DNA Methylation

The DNA methylation state of MBCs is controlled by DNA methyltransferases (DNMTs). In Shaknovich et al. [13], a general hypomethylation state was found in GC B cells and DNMT1 was presented as an essential regulator of the GC formation. This study was challenged by the lethality of DNMT1 knockout mice, as its disruption affected other cell types in development. Addressing this issue, through tissue-specific downregulation of DNMT1, the authors identified DNMT1 to be necessary for the primary follicle to functional GC B cell transition [13]. Analogously, ten-eleven-translocation (TET) proteins (TET1, TET2, and TET3) were essential for DNA demethylation at GC B cells, allowing for AID expression and functional antibody maturation, key for the subsequent B cell differentiation [16]. Nevertheless, the need of DNMT1 and TET proteins on GC-dependent memory formation was not studied, leaving a major knowledge gap. A recently proposed chromatin remodeler is lymphoid-specific helicase (HELLS), which together with DNMT1 was crucial to maintain the GC B cell program and prevent MBC selection [14]. In fact, an accelerated pre-MBC phenotype is observed upon HELLS downregulation and DNMT1 inhibition, suggesting the role of HELLS in controlling the MBC fate determination [14]. Among the upregulated genes, two relevant MBC markers were observed: hematopoietically-expressed homeobox (HHEX) and chemokine receptor 6 (CCR6) [5, 9, 14]. Conversely, epigenomic profiling studies demonstrated that DNMT3a is present at relatively elevated levels at the MBC and PC states when compared to GC B cells, which express higher levels of DNMT1 and DNMT3b [15]. This study also presented striking evidence for GC B cell methylation reprograming, maintaining low levels of de novo methylation reestablishment upon MBC and PC differentiation when compared to naïve B cells [15]. In this manner, the methylation profiles of MBCs and PCs were closer to each other [15], suggesting a less complex transition for MBC differentiation upon pathogen reencounter. Together, this data supports the existence of a strict epigenetic switch between naïve and activated B cell states, and a poised switch among GC B cells, MBCs, and PCs. Furthermore, when DNMT3a and DNMT3b were conditionally knocked out in mice, GC PC differentiation was evidently repressed [17]. DNMTs 3a and 3b are particularly important for de novo DNA methylation, possibly providing the necessary plasticity for GC B cell fate determination [17, 18]. Considering that the combined disruption of DNMT3a and DNMT3b may have triggered a stronger repressor effect on PC differentiation, this study lacked the separate deletion of each individual methyltransferase, and did not account for the high expression of DNMT3a at the MBC pool, as previously described [15, 17]. In summary, these studies support the idea of a transcriptional methylation switch between DNMT1, DNMT3b, and potentially HELLS at the GC, and DNMT3a at MBC and PC selection. Consequently, the DNA methylation state of GC B cells and MBCs provides further comprehension of the differential B cell fate epigenetic program.

### 1.3. Epigenetic Control of B Cell Memory by Histone Modifications

In contrast to DNA methylation, more is known about the regulation of the MBC state and differentiation by histone modifications. A dynamic balance between activating and repressive histone marks has been argued to control chromatin for accessibility and gene transcription at each B cell differentiation state [42].

Histone methylation of differentiated B cells is controlled by enhancer of zest 2 (EZH2) in a poised manner, and putatively regulated by other histone lysine methyltransferases and demethylases. As part of the polycomb repressive complex 2 (PRC2), the histone modifier EZH2 is an important epigenetic regulator of the GC reaction and repressor of the B cell transcriptional program [19–21]. In this manner, EZH2 has been widely described to promote histone 3 lysine 27 trimethylation (H3K27me3), a repressive histone modification closing the chromatin at the GC state [19, 21]. Thus, in normal physiological conditions, GC B cells express higher levels of EZH2 with respect to differentiated MBCs and PCs [19]. Upon EZH2 gene disruption, GC formation was impaired and early B cell differentiation resulted in rapid lymphomagenesis and fewer functional MBCs [19, 20]. Additionally, EZH2 epigenetically enables the high proliferation rate of GC B cells by silencing cell cycle inhibitors, such as the cyclin-dependent kinase inhibitor CDKN1A, a critical feature of rapidly dividing lymphocytes [22]. In conjunction, these studies suggested a role for EZH2 in establishing and maintaining the GC B cell state while inhibiting MBC and PC differentiation. Mechanistically, EZH2 was proposed to regulate the GC B cell transcriptional program in cooperation with B cell lymphoma 6 protein (BCL6), the “master” transcription factor of GC B cells [23]. Analogously, BCL6 is known to provide a multi-layer, structural control of the GC B cell program, later discussed in this review [43]. More evidence supporting the EZH2 control over B cell differentiation came from differential accessible region analyses. Scharer et al. [24] used ChIP-seq and ATAC-seq to reveal that a closed chromatin state and an increased H3K27me3 profile is present at more than 300 promoter regions in early B cell stages. Interestingly, this repressive epigenetic profile was lost upon B cell differentiation [24]. EZH2 histone methylation was shown to prevent the transcription of important regulators of B cell differentiation, including two key PC fate markers: B lymphocyte-induced maturation protein 1 (BLIMP1) and interferon-regulatory factor 4 (IRF4) [5, 19, 24]. Therefore, by locking activated B cells to the GC, EZH2 regulates the bivalent switch of B cell fate determination at the GC. In addition, defects in lysine methyltransferases (KMTs) and lysine demethylases (KDMs) have been associated with impaired B cell memory formation and lymphomagenesis [25–27]. The histone 3 lysine 4 (H3K4) methyltransferase KMT2D was first described in two independent studies to counterbalance the repressive GC B cell epigenetic program by accelerating GC formation and promoting B cell development [25, 26]. KMT2D mediates monomethylation of histone H3 lysine 4 (H3K4me), an activating mark at enhancers. Opposite to the repressive H3K27me3 mark, histone 3 lysine 4 mono- and trimethylation (H3K4me and H3K4me3) activate the B cell differentiation program [44]. For instance, this occurs by upregulating B cell antiapoptotic factors, such as B cell lymphoma proteins 2 (BCL2) and 2L1 (BCL2L1), as well as cell cycle regulators including cyclin-dependent kinase 6 (CDK6) [25]. Intriguingly, KMT2D expression only decreased in fully differentiated PCs, implying a continuous role at GC B cell and MBC states [26]. Later transcriptomic analyses confirmed the lymphomagenic potential of the DNMTs EZH2 and KMT2D, and identified an analogous tumor suppressor role for the lysine demethylase KDM4C [27]. In the context of immunological memory, KDM4C is not yet functionally characterized [27]. Collectively, these studies demonstrate that the loss of epigenetic control at GCs leads to lymphomagenesis characterized by an impaired MBC and PC differentiation. For this reason, it has been challenging to fully comprehend the functional roles of DNMTs and demethylases in MBC fate determination. Thus far, while EZH2 is regarded as the major epigenetic regulator of the GC B cell program, other methylation regulators, such as KMT2D and KDM4C, should be further investigated to discover the role of histone modifications in B cell memory formation.

In a similar manner, histone acetyltransferases and deacetylases have been described to regulate the GC B cell, MBC, and PC differentiation states. This includes histone deacetylase 3 (HDAC3), the p300 histone acetyltransferase, CREB-binding protein (CREBBP) and monocytic leukemia zinc finger (MOZ). HDAC3 removes acetyl groups from histones (e.g. H3K27ac), promoting chromatin condensation. Comparable to the H3K4 methylation mark, histone 3 lysine 9 acetylation (H3K9ac) and histone 3 lysine 27 acetylation (H3K27ac) are activating epigenetic marks [44]. Thus, deacetylases as HDAC3 repress chromatin opening and acetyltransferases like p300, CREBBP, or MOZ promote it. Recruited by BCL6 in complex with SMRT (silencing mediator for retinoic acid receptor and thyroid hormone receptor), the H3K27 deacetylase HDAC3 was proposed to repress PC determination by silencing major B cell differentiation gene enhancers including *Prdm1*, the gene coding for BLIMP1 [28, 29]. Its inhibitory mechanism was suggested to be in complex with BACH2 (broad complex-tramtrack-bric a brac and Cap'n'collar homology 2), a major regulator of the MBC program [9, 29]. Thus, BACH2 was proposed to interact with HDAC3 to inhibit BLIMP1 expression and hence, prevent PC fate determination while maintaining the GC B cell and MBC states [29]. Interestingly, this effect was then antagonized by the H3K27 acetyltransferase p300, inducing enhancer reactivation [28]. Moreover, there is evidence for a structural and functional costimulatory similarity between p300 and the histone acetyltransferase CREBBP [30]. Thus, deregulated expression patterns and similar genetic defects in any of these factors resulted in common types of B cell transformations [30]. Noting the importance of maintaining the histone acetylation balance in B cells, mice with impaired CREBBP/p300 acetylation resulted in a rapid onset of B cell lymphomas [31]. Consequently, still pending further research about these factors combined, p300 and CREBBP can be associated to counteracting the GC B cell histone deacetylation patterns, promoting B cell differentiation, and suppressing malignant transformations in B cells. Accordingly, the histone acetyltransferase MOZ was reported to regulate the GC formation and control MBC differentiation by promoting H3K9ac [32]. When MOZ was conditionally deleted, BCL6 expression decreased, and two classes of premature unswitched IgM and class-switched IgG1 MBCs were produced [32]. Limiting this study, the authors argued that the IgM MBC group later developed into GC B cells at secondary immune responses [32]. Nevertheless, the GC formation was altered in the first place by deleting MOZ, and recent in vivo fate-mapping studies have elucidated that it is in fact naïve B cells forming new GCs during secondary responses [45]. Hence, the clonal dynamics of MOZ-deficient premature MBCs should be further explored using other tools, such as the fluorescently-labeled AID-expressing cells from Confetti mice [45] or novel spatial transcriptomics methods, such as spatial V(D)J [46]. Altogether, the H3K27 acetylation switch is then maintained by a dynamic interplay between HDAC3, controlling the GC B cell state, and p300 jointly with CREBBP, promoting B cell differentiation. Thereafter, the inclination towards the MBC fate is proposed to be reinforced by MOZ-dependent H3K9ac.

In sum, different histone modifications and regulatory factors establish a tight level of control over the B cell epigenetic program. Evidence supporting a poised pattern of histone modifications suggests the existence of a dynamic epigenetic switch regulating transcription in GC B cells, MBCs, and PCs.

### 1.4. Epigenetic Control of B Cell Memory by Non-Coding RNAs (ncRNAs)

The posttranscriptional regulation of the B cell memory program is mediated by ncRNA, with micro RNAs (miRNA or miR) and long ncRNAs (lncRNA) representing the most prominent examples. Nevertheless, other types of ncRNAs and RNA-binding proteins could also play a role in regulating B cell memory formation. In sum, miRNAs are small RNA molecules inducing transcriptional repression of partially complementary messenger RNA (mRNA) transcripts [47]. At the GC, miR-155 was extensively argued to control the GC B cell state and affinity maturation [2, 33, 47]. However, its governing control over the GC B cell program was not understood until Basso et al. [34] demonstrated that by repressing miR-155, BCL6 was hampering the inhibition of miR-155 towards AID. With less evidence, miR-361 was also proposed to regulate AID expression mediated by BCL6 [34]. Furthermore, miR-155 upregulation in PBs was required for GC-dependent B cell differentiation, proliferation, and survival [35]. In conjunction, this data suggests that miR-155 plays an important role in establishing the GC reaction, and allowing hypermutated GC B cells from the light zone to escape the GC and differentiate into MBCs and PCs. On the other hand, miR-125b is a miRNA studied to impede GC B cells to differentiate into PCs at the dark zone by repressing BLIMP1 and IRF4 transcripts [36]. Notwithstanding this study shows that miR-125b is expressed in low levels at the MBC stage, BLIMP1 and IRF4 levels are not increased in this cell type [36]. Hence, miR-125b must not be the only posttranscriptional repressor of BLIMP1 and IRF4 mRNAs. Promoting B cell survival, miR-15a and miR-16, two miRNAs targeting BCL-2 and other antiapoptotic factors, have been shown to be upregulated in the GC but downregulated in MBCs [37]. Thus, mutations in these two miRNAs lead to dysregulated B cell growth, differentiation, and lymphomagenesis [37]. When it comes to miRNAs involved in MBC phenotype stability, an integrative transcriptomics analysis resolved miR-181 downregulation to permit MBC gene signature expression [38]. Intriguingly, this study revealed a differential miRNA expression at class-switched MBCs and a transitory general epigenetic profile at unswitched MBCs [38]. For further reading, Fuertes et al. [47] recently reviewed the state-of-the-art of miRNAs regulating the GC reaction, including supplementary negative regulators of MBC and PC differentiation. Thereafter, there are putative miRNA regulators of B cell memory formation with insufficient evidence that should be further studied. This includes the yet poorly investigated miRNAs miR-23b, miR-28, miR-29c, miR-30a, miR-146, miR-199, miR-217, miR-223, miR-342, and let-7e [2, 11, 38, 47]. The other examined type of posttranscriptional control of B cell memory is by lncRNA gene expression modulation. The lncRNAs TCL6, RP11-164H13.1, and TUNAR have been found downregulated at class-switched MBCs [38]. The expression of these lncRNAs was correlated to the chromatin accessibility and gene transcription of the immunoglobulin heavy locus (IGH) genes [38]. Complementary to this finding, AL928768, COPDA1, and RP11-731F5.1 were overexpressed in class-switched MBCs, the three being lncRNAs encoded upstream or downstream the loci of IGHA1, IGHG2, and IGHE [38]. Among other possible functions, lncRNAs can regulate transcription of the IGH gene and influence B cell memory by controlling the expression of the different antibody isotypes in MBCs. Taking all these findings together, B cell memory formation is tightly controlled by miRNAs and lncRNAs, making the posttranscriptional regulation an essential regulatory step to establish immunological memory.

### 1.5. Epigenetic Control of B Cell Memory by Chromatin Topology

The last known level of epigenetic regulation of B cell memory formation is through chromatin rearrangements and higher-level structural accessibility of the chromatin. In Bunting et al. [43], a multi-layered epigenetic reprograming is proposed to coordinate the transition from naïve to GC B cells. Studying the changing conformation of the chromatin, the BCL6 locus was found to be central not only for transcription of GC B cell factors, but also to control promoter–promoter interactions, promoter-enhancer interactions, three-dimensional gene looping, and DNA-histone code reprograming [43]. As these structural epigenetic marks were required for functional GC formation, this study shed a light to the relevance of reorganizing the genome to control the transition between B cell epigenetic programs. Thus, similar three-dimensional structural changes must occur to establish and stabilize the MBC phenotype. From this standpoint, few chromatin remodeling factors have been described. Bromodomain and WD repeat-containing 1 (BRWD1) is a chromatin reader known to coordinate the chromatin topology transition between early and late B cell development stages [39]. Necessary to initiate the GC reaction and transition among GC B cell states, BRWD1 is upregulated at the GC, influences B cell affinity maturation, and restrains further B cell proliferation [40]. However, BRWD1 deletion did not significantly influence MBC and PC differentiation [40]. Being only preliminary data available on BRWD1 and B cell memory formation, this study does not support the influence of BRWD1 in MBC fate determination. Conversely, often mutated in diffuse large B cell lymphomas, AT rich interactive domain 1A (ARID1A) can directly bind to DNA and proteins to orchestrate the MBC fate program [31, 41]. Loss of ARID1A led to an impaired GC formation and an early GC B cell exit and differentiation into preMBCs [41]. ARID1A was found to regulate the GC B cell differentiation transition by interacting with the transcription factors PU.1 and NF-kB and accordingly, upregulating MBC signature genes, such as BACH2, HHEX, and KLF2 while downregulating BCL6 [41]. Collectively, a multi-tier reorganization of the genome's topology is required to allow for such complex transcriptional transitions between B cell states. Subsequently, direct chromatin remodelers are being inquired to control the formation of immune memory in B cells.

### 1.6. Poised Model of Epigenetic Reprograming in B Cell Memory Formation

**Figure 1:**
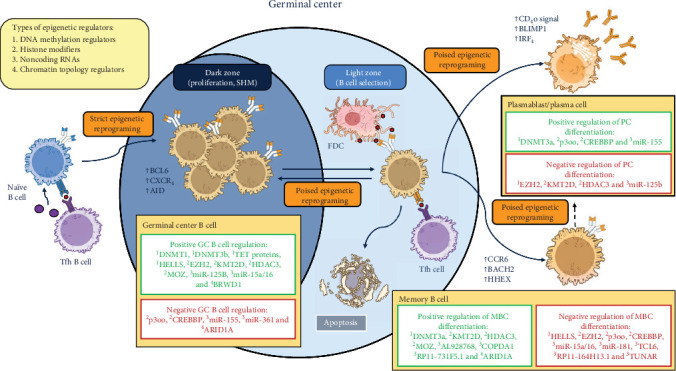
Poised model of epigenetic reprograming in B cell memory formation. Representative model of the main transcription, epigenetic, and posttranscriptional regulators of memory B cell differentiation. Positive and negative epigenetic regulators of germinal center B cells, memory B cells, and plasma cells establish a dynamic environment, where transcription regulator genes are expressed in a poised manner. As such, chromatin modifiers, posttranscriptional regulators and a three-dimensional reorganization of the genome promotes rapid transcriptional changes and the formation of a dynamic switch. The presented model focuses on the epigenetic regulators establishing and repressing the memory B cell phenotype. Four different types of epigenetic regulators are presented, labeled 1–4 as stated in the figure legend. AID, activation-induced cytidine deaminase; ARID1A, AT-rich interactive domain 1A; BACH2, broad complex-tramtrack-bric a brac and Cap'n'collar homology 2; BCL6, B cell lymphoma 6; BLIMP1, B lymphocyte-induced maturation protein 1; BRWD1, bromodomain and WD repeat-containing 1; CCR6, chemokine receptor 6; CREBBP, CREB-binding protein; COPDA1, chronic obstructive pulmonary disease A1; CXCR4, C-X-C chemokine receptor type 4; DNMT1, DNA methyltransferase 1; DNMT3a, DNA methyltransferase 3a; DNMT3b, DNA methyltransferase 3b; EZH2, enhancer of zest 2; GC, germinal center; HDAC3, histone deacetylase 3; HELLS, lymphoid-specific helicase; HHEX, hematopoietically-expressed homeobox; IRF4, interferon-regulatory factor 4; KMT2D, lysine methyltransferase 2d; MBC, memory B cell; miR, micro-RNA; MOZ, monocytic leukemia zinc finger; PC, Plasma cell; TCL6, T-cell leukemia/lymphoma 6; TET proteins, ten-eleven-translocation proteins; Tfh cell, T follicular helper cell; TUNAR, TCL1 upstream neural differentiation-associated RNA. Figure created by the author, inspired by references [5] and [38]. Illustrations taken from NIAID NIH BIOART Source (bioart.niaid.nih.gov/bioart/).

A dynamic epigenetic landscape orchestrates the transcriptional program of GC B cells, MBCs, and PCs in a poised manner to generate B cell immune memory. While the transcriptional control of MBC differentiation has been widely investigated and reviewed, the major epigenetic regulators of B cell memory formation are poorly integrated to the current paradigm [9]. Combining the discussed epigenetic marks into the present models of MBC differentiation (presented in [5] and [9]), epigenetics provides the necessary layer of control governing MBC cell fate determination at the GC (Figure 1). This is achieved by creating a bivalent environment, where survival genes, proliferation genes, and transcription regulators can be switched on and off upon activator or repressive signals from the environment. In this context, bivalent chromatin refers to regions marked by both active (e.g. H3K4me3) and repressive (e.g. H3K27me3) epigenetic marks, keeping genes transcriptionally silent but primed for activation. For instance, the strength of Tfh cell help and BCR signaling are important signals capable to switch the poised balance and induce apoptosis if null, MBC differentiation if low, GC reentry if intermediate and PC differentiation if strong [9, 48]. Shinnakasu et al. [48] identified BACH2 expression to be inversely correlated with the level of Tfh cell help, suggesting this transcriptional repressor of the PC program to be a key regulator of MBC differentiation. With this evidence, an affinity-dependent instructive model of MBC fate determination was proposed but no subsequent epigenetic remodeling was considered [5, 9, 48]. As previously argued, BACH2 acts in complex with HDAC3 to silence BLIMP1 expression and prevent PC differentiation [29]. Furthermore, BACH2 expression is regulated itself by the chromatin remodeler ARID1A [41]. In addition, Tfh cells not only support B cells by the strength of the CD40L-CD40 and TCR-MHCII interaction, but they also secrete cytokines and extracellular vesicles containing miRNAs to regulate B cell survival, differentiation, and antibody CSR [11, 49]. Thus, poised genes may lead to different B cell fates based on the strength of Tfh cell and BCR signaling. Nonetheless, this model appeared to be incomplete upon the finding of low and high affinity MBCs and PBs secreted at early stages of the GC reaction [5, 9, 50]. Moreover, the time spent in the GC by a recycling B cell correlated with the general increase in the affinity of its BCR upon export as MBC or PC [50]. In a similar way, the time-dependent stochastic model of MBC differentiation complements the proposed model of poised genes inducing B cell memory formation. In early GCs, there is a wide clonal diversity of GC B cells undergoing affinity maturation and clonal selection [5]. In the same manner, different epigenetic programs must be present at the GC. Then, GC B cells are allowed to stay at the GC, undergo apoptosis or exit the GC as MBC or PC depending on the state of its epigenetic profile. As the B cell spends time in the GC, random engagement with antigen and Tfh cells, as well as casual interactions with the environment, will induce the accumulation of epigenetic marks. For example, if H3K27me3 marks are accumulated in B cell differentiation genes by EZH2 in cooperation with BCL6, the B cell will stay at the GC. On the contrary, if miR-181 is downregulated and the chromatin remodeler ARID1A is overexpressed, the MBC state will be promoted. Presented in Figure 1, a poised model of epigenetic reprograming controlling MBC differentiation is proposed for the first time. On that account, a dynamic regulatory environment integrates the transcriptional, posttranscriptional, and epigenetic control of B cell memory formation. Nevertheless, the extent of this type of poised epigenetic control over the selection steps at the GC is still to be determined and should not only be studied for poised genes and their expression. Beyond bivalent genes, poised promoters and enhancers marked by activator and repressive marks may also contribute to epigenetic flexibility during MBC differentiation.

## 2. Conclusion

In the recent years, biological investigations inquiring the dynamics of GCs have grounded evidence for multiple possible explanations of B cell fate determination and B cell memory formation [5, 9, 51]. However, there is no current model considering the epigenetic state and reprograming of each B cell differentiation stage. As discussed in this essay, MBCs are products of the GC reaction and present a different transcriptional and epigenetic profile with respect to GC B cells and PCs. While this review focuses on GC-derived MBCs, it is important to note that GC-independent MBC populations exist and may utilize distinct epigenetic pathways, warranting further investigation. Beyond selection at the GC, recent findings suggest the involvement of a bivalent epigenetic switch in GC B cells cycling between the dark and light zones [52, 53]. An observed decrease in SHM during the GC burst phase, and potentially during selection, as well as the reduction in class switching, may be driven by the epigenetic reprograming mechanisms outlined here [52, 53]. These changes likely modulate accessibility and activity of key loci involved in SHM and CSR in a poised manner, linking epigenetic control directly to functional B cell diversification dynamics. In sum, MBC formation requires a tight and multi-layer epigenetic regulation to allow for coordinated chromatin accessibility, transcriptional reprograming, and effective posttranscriptional regulation. Epigenetic factors including DNMTs, histone modifiers, ncRNAs, and other chromatin readers and remodelers all influence the formation of immunological memory. Thus, these regulators function in coordination to allow for a dynamic and reversible MBC program. Considering these findings, a novel model has been put forth that situates epigenetics at the governance of B cell memory formation. Beyond memory formation, the role of epigenetics in the maintenance of B cell memory and MBC reactivation still remains unclear and lesser evidence is present [51]. Comprehending these mechanisms better would allow for improved vaccination and therapeutical approaches.

## Figures and Tables

**Table 1:** Epigenetic regulators of B cell memory formation.

Regulators	Type	Putative function(s)	References
DNA methylation regulators			
DNMT1	DNA demethylation	Maintenance of the germinal center B cell hypomethylated state	[13–15]
TET Proteins	DNA demethylation	Promotion of AID expression at the germinal center	[16]
DNMT3a	*De novo* DNA methylation	Activating switch at memory B cell and plasma cell differentiated states	[15, 17, 18]
DNMT3b	*De novo* DNA methylation	Contribution to the regulation of the germinal center B cell program	[15, 17, 18]
HELLS	DNA methylation	Control of memory B cell markers at the germinal center (including HHEX and CCR6)	[14]
Histone modifiers			
EZH2	Histone methyltransferase	Regulation of germinal center B cells by repressing B cell differentiation transcription factors (including BLIMP1 and IRF4)	[19–24]
KMT2D	Histone methyltransferase	Promotion of germinal center formation and activation of B cell development	[25–27]
KDM4C	Histone demethylase	Repression of B cell lymphoma formation, function not yet known	[27]
HDAC3	Histone deacetylase	Together with BCL6-SMRT complexes and BACH2, silencing of plasma cell differentiation enhancers (including BLIMP1)	[28, 29]
p300	Histone acetyltransferase	Balancing HDAC3 transcriptional repression by reactivating B cell differentiation enhancers	[28, 30, 31]
CREBBP	Histone acetyltransferase	Counteraction of repressive histone deacetylations and suppressor of lymphomagenesis	[31]
MOZ	Histone acetyltransferase	Endorsement of memory B cell differentiation and germinal center maintenance	[32]
Noncoding RNAs			
miR-155	Micro-RNA	Germinal center formation and AID control, B cell differentiation at light zone and plasmablast survival	[33–35]
miR-361	Micro-RNA	Regulation of AID expression at germinal center B cells	[34]
miR-125b	Micro-RNA	Regulation of the germinal center dark zone B cells by inhibiting BLIMP1 and IRF4	[36]
miR-15a/16	Micro-RNA	Repression of B cell anti-apoptotic genes (including BCL2 and CDKs), downregulated in memory B cells	[37]
miR-181	Micro-RNA	Micro RNA, promotion of the memory B cell transcriptional program, downregulated in memory B cells	[38]
TCL6, RP11-164H13.1 and TUNAR	Long noncoding RNAs	Downregulated in isotype-switched memory B cells, putative regulation of the *IGH* gene transcription by controlling chromatin accessibility	[38]
AL928768, COPDA1and RP11-731F5.1	Long noncoding RNAs	Upregulated in class-switched memory B cells, putative regulation of transcription at the *IGHA1*, *IGHG2*, and *IGHE* loci	[38]
Chromatin topology regulators			
BRWD1	Chromatin reader and remodeler	Establishment of the germinal center reaction and early B cell development	[39, 40]
ARID1A	Chromatin reader and remodeler	Coordinator of structural rearrangements leading to memory B cell gene upregulation and differentiation	[41]

*Note:* Table created by the author.

Abbreviations: AID, activation-induced cytidine deaminase; ARID1A, AT-rich interactive domain 1A; BACH2, broad complex-tramtrack-bric a brac and Cap'n'collar homology 2; BCL6, B cell lymphoma 6; BLIMP1, B lymphocyte-induced maturation protein 1; BRWD1, bromodomain and WD repeat-containing 1; CCR6, chemokine receptor 6; CDK, cyclin-dependent kinase; CREBBP, CREB-binding protein; COPDA1, chronic obstructive pulmonary disease A1; DNMT1, DNA methyltransferase 1; DNMT3a, DNA methyltransferase 3a; DNMT3b, DNA methyltransferase 3b; EZH2, enhancer of zest 2; HDAC3, histone deacetylase 3; HELLS, lymphoid-specific helicase; HHEX, hematopoietically-expressed homeobox; IGH, immunoglobulin heavy locus; IRF4, interferon-regulatory factor 4; KDM4C, lysine demethylase 4c; KMT2D, lysine methyltransferase 2d; miR, micro RNA; MOZ, monocytic leukemia zinc finger; SMRT, silencing mediator for retinoic acid receptor and thyroid hormone receptor; TCL6, T-cell leukemia/lymphoma 6; TET proteins, ten-eleven-translocation proteins; TUNAR, TCL1 upstream neural differentiation-associated RNA.

## Data Availability

The author has nothing to report.

## Nomenclature

AID: Activation-induced cytidine deaminase
ARID1A: AT-rich interactive domain 1A
BACH2: Broad complex-tramtrack-bric a brac and Cap'n'collar homology 2
BRWD1: Bromodomain and WD repeat-containing 1
BCR: B cell receptor
BLIMP1: B lymphocyte-induced maturation protein 1
BCL6: B cell lymphoma 6
BCL2: B cell lymphoma 2
BCL2L1: B cell lymphoma 2L1
CCR6: Chemokine receptor 6
CSR: Class switch recombination
CREBBP: CREB-binding protein
CDK6: Cyclin-dependent kinase 6
CXCR4: C-X-C chemokine receptor type 4
DNMTs: DNA methyltransferases
EZH2: Enhancer of zest 2
GC: Germinal center
HHEX: Hematopoietically-expressed homeobox
H3K4: Histone 3 lysine 4
H3K4me: Histone 3 lysine 4 monomethylation
H3K4me3: Histone 3 lysine 4 trimethylation
H3K9ac: Histone 3 lysine 9 acetylation
H3K27ac: Histone 3 lysine 27 acetylation
H3K27me3: Histone 3 lysine 27 trimethylation
HDAC3: Histone deacetylase 3
IRF4: Interferon-regulatory factor 4
IGH: Immunoglobulin heavy chain genes
lncRNA: Long noncoding RNA
HELLS: Lymphoid-specific helicase
KDM: Lysine demethylase
KMT: Lysine methyltransferase
MBC: Memory B cell
mRNA: Messenger RNA
miRNA: Micro RNA
MOZ: Monocytic leukemia zinc finger
ncRNA: Noncoding RNA
PB: Plasmablast
PC: Plasma cell
PRC2: Polycomb repressive complex 2
RAG1 and RAG2: Recombination-activating genes
SMRT: Silencing mediator for retinoic acid receptor and thyroid hormone receptor
SHM: Somatic hypermutation
Tfh cell: T follicular helper cell
TET: Ten-eleven-translocation proteins
V(D)J: Variable, diversity, and joining immunoglobulin genes.
